# Association of medullary sponge kidney and hyperparathyroidism with *RET* G691S/S904S polymorphism: a case report

**DOI:** 10.1186/s13256-018-1736-6

**Published:** 2018-07-09

**Authors:** Muhammad Usman Janjua, Xiao-dan Long, Zhao-hui Mo, Chang-sheng Dong, Ping Jin

**Affiliations:** 1 Department of Endocrinology, The Third Xiangya Hospital, Central South University, Tongzipo Road, Changsha, 410007 Hunan Province People’s Republic of China; 2 Department of Anesthesia, The Affiliated Tumor Hospital of Xiangya Medical School of Central South University, Changsha, 410007 Hunan China

**Keywords:** Medullary sponge kidney, Hyperparathyroidism, *RET*, Polymorphism

## Abstract

**Background:**

Medullary sponge kidney is a rare renal malformation, which usually manifests as nephrocalcinosis, renal tubular acidosis, and recurrent urinary tract infections. Medullary sponge kidney is often associated with renal developmental anomalies and tumors, and its exact pathogenesis is not yet clearly explained. Given the key role of the interaction of glial cell line-derived neurotrophic factor gene, *GDNF*, and the “rearranged during transfection” proto-oncogene, *RET*, in kidney and urinary tract development, variations in these genes are proposed to be candidates for medullary sponge kidney. Hyperparathyroidism is observed in a few patients with medullary sponge kidney, but the exact pathogenesis of this association is unknown. This case report highlights the coexistence of these two conditions associated with *RET* polymorphism, which contributes toward the understanding of the pathogenesis of medullary sponge kidney.

**Case presentation:**

A 52-year-old Chinese woman with recurrent renal stones presented to our hospital. Subsequently she was diagnosed as having medullary sponge kidney and tertiary hyperparathyroidism and underwent parathyroidectomy. Genomic DNA was isolated from lymphocytes and the *GDNF* and *RET* genes were determined by Sanger sequencing. Two *RET* polymorphisms were found in our patient, one was nonsynonymous c.2071G>A (G691S; rs1799939) located in exon 11, the other was synonymous c.2712C>G. (p.S904S; rs1800863) located in exon 15.

**Conclusions:**

We demonstrated a case of medullary sponge kidney combined with tertiary hyperparathyroidism, which contributes to further understanding of the pathogenesis of this disease. Besides, we also found *RET* G691S/S904S polymorphism in this patient, but additional studies are required to explore the role of the *RET* gene in medullary sponge kidney with hyperparathyroidism.

## Background

Medullary sponge kidney (MSK) is a nephropathy which usually manifests as nephrolithiasis, renal tubular acidosis, concentration defects, medullary cystic dilatations, and recurrent urinary tract infections. Its incidence is 1/20,000 to 1/5000 in the general population, 3 to 5% in patients with kidney stones, and up to 20% in patients with recurrent kidney stones [1]. MSK generally occurs sporadically, but an apparently autosomal dominant inheritance has also been observed in familial cases [2]. MSK is often associated with renal developmental anomalies and tumors, such as Wilms tumor, horseshoe kidney, and contralateral congenital small kidney, which supports the conviction that it is a developmental disorder [3]. Nephrogenesis depends on reciprocal inductive interactions between the ureteric bud and the metanephric blastema. During renal organogenesis, glial cell line-derived neurotrophic factor (GDNF) produced by the metanephric blastema, and its receptor “rearranged during transfection” (RET) on the ureteric bud, induces ureteric bud outgrowth and branching from Wolff’s duct [4, 5]. Thus, some speculated that variants of *GDNF* and *RET* genes, which play a key role in kidney and urinary tract development, maybe reasonable candidates for MSK. Hyperparathyroidism is observed in a few patients with MSK, but the exact pathogenesis of this association is unknown [3, 6]. In the current study, we present a case of MSK accompanied with tertiary hyperparathyroidism in whom we analyzed the *GDNF* and *RET* gene variations, which contributes to further understanding of the pathogenesis of this disease.

## Case presentation

**Fig. 1:**
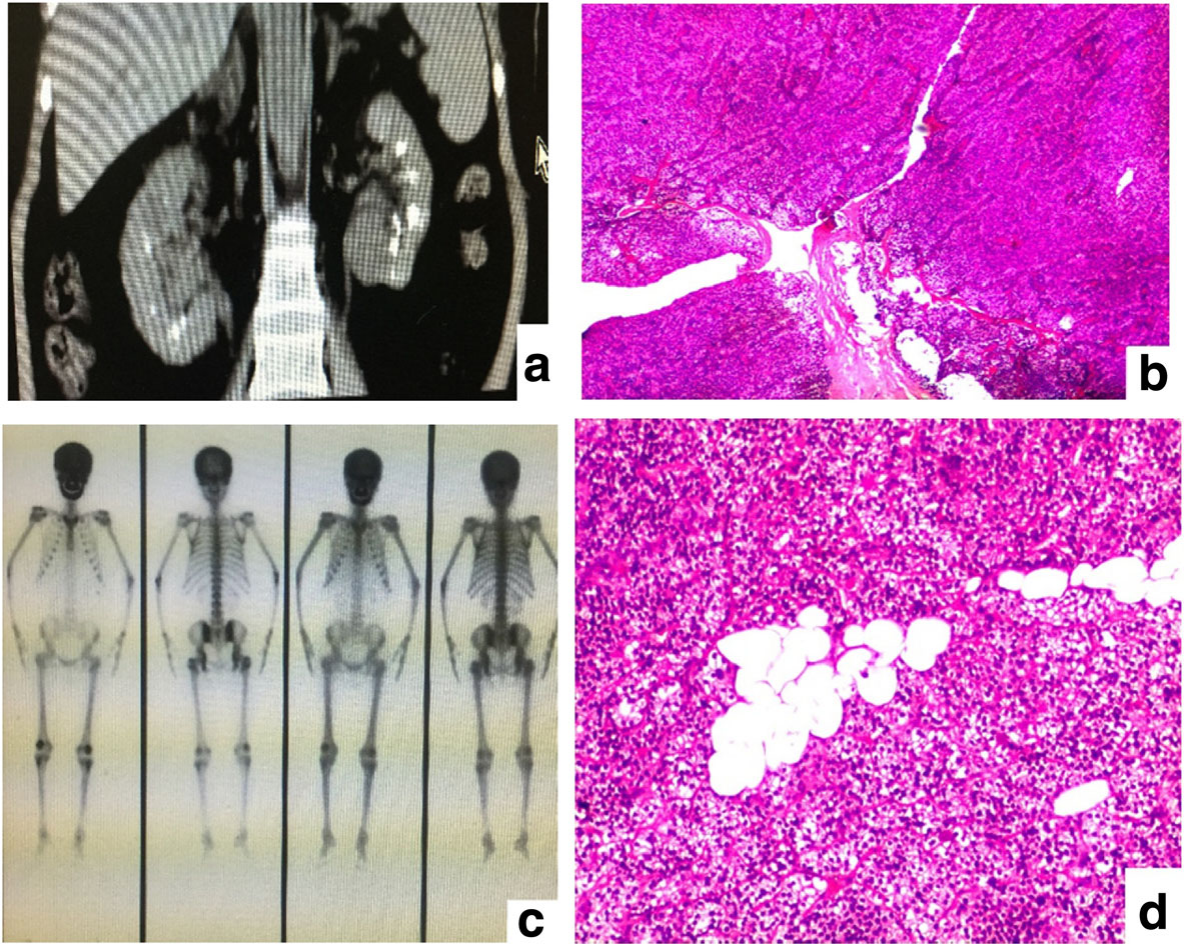
**a** Computed tomography of the kidney showed a great amount of dotted and patchy compact shades at deep medullary sections of both kidneys, which indicated medullary sponge kidney and polycystic kidney disease. **b** Histological view of resected right parathyroid tissue showed adenomatous hyperplasia of parathyroid glands stain at magnification × 100 (f). **c** A ^99m^Tc bone scan revealing increased uptake. **d** The histological examination of the resected left parathyroid tissue showed nodular hyperplasia and active regional cellular hyperplasia which was in accordance with parathyroid nodular hyperplasia

A 52-year-old Chinese woman presented to our hospital in September 2012 with a complaint of recurrent renal stones for 6 years. The renal stones were first discovered at a local hospital 6 years ago and then she underwent bilateral ureteroscopic lithotomy and ultrasonic lithotripsy several times, but the calculus still relapsed and her serum creatinine gradually increased to 240 to 300 umol/L. She was a teacher living with her husband and two children, all healthy, in a small city. She did not smoke tobacco or consume alcohol. She had no significant past medical history, she had not suffered from any infectious disease, and she did not suffer from any chronic illness. There was no family history of similar disease or any other chronic illness. There was no history of allergy to any food or drugs. On physical examination: temperature (T) 37.5 °C, pulse (P) 82/minute, respiratory rate (R) 20/minute, blood pressure (BP) 130/80 mmHg, weight (W) 51 kg, and height (H) 154 cm. Respiratory movement and cardiac examination were normal. On abdominal examination no masses or tenderness were noted on both light and deep palpation. Her liver and spleen were not palpable. A sensory and motor system examination did not reveal any abnormality. Her neurological reflexes were normal. A routine urine examination showed: white blood cell 87/hpf (WBC2+), red blood cell 34/hpf (RBC2+), and pH 7. 0. A routine blood test showed: hemoglobin (Hb) 95 g/L. Her serum potassium was 3.2 mmol/L, calcium 2.92 mmol/L, phosphate 1.3 mmol/L, carbon dioxide combining power (CO_2_CP) 17.8 mmol/L, creatinine 249 μmol/L, serum parathyroid hormone (PTH) 1147 pg/ml (Table 1), 25-hydroxyvitamin D3 level 42.98 nmol/L with reference range (RR) of 47.7–144 nmol/L, 24-hour urinary calcium 8.98 mmol/day (RR, 2.5–7.5 mmol/day), and 24-hour urine potassium 26.8 mmol/day. Liver and thyroid functions were all normal. Urine culture showed the presence of *Escherichia coli*. Renal ultrasound showed multiple stones, cysts, and calcified lesions. Computed tomography (CT) of her kidney showed MSK and polycystic kidney disease (Fig. 1a). Ultrasonography of her thyroid and parathyroid showed two hypoechoic nodules in the right lobe of her thyroid with the larger one of 26 × 18 mm. She was diagnosed as having MSK complicated with tertiary hyperparathyroidism and underwent right parathyroidectomy on September 18, 2012. Histopathologic examination revealed adenomatous hyperplasia of parathyroid glands (Fig. 1b). After surgery, her serum potassium was 3.3 mmol/L, calcium 2.81 mmol/L, phosphate 1.01 mmol/L, CO_2_CP 16.1 mmol/L, PTH 742.5 pg/ml, and serum creatinine 231 μmol/L (Table 1). The oral form of iron and calcitriol was administered.

**Table 1:** Biochemical characteristics of the patient

	6 Sep 2012	20 Sep 2012	4 Mar 2013	28 Mar 2013	7 Jul 2016	Reference ranges
Calcium (mmol/L)	2.92	2.81	2.83	2.25	2.09	2.08–2.8
Phosphate (mmol/L)	1.3	1.01	1.21	1.07	2.37	0.9–1.34
PTH (pg/mL)	1147	742.5	1378	47.8	766.4	15–65
Potassium (mmol/L)	3.2	3.3	3.1	3.7	4.0	3.5–5.2
CO_2_CP (mmol/L)	17.8	16.1	19.7	16.8	15.6	23–29
CRE (umol/L)	249	231	265	257	766	45–104
Urine PH	7.0		7.5		7.0	

*CO*_*2*_*CP* Carbon dioxide combining power, *CRE* creatinine, *PTH* parathyroid hormone

In March 2013, 6 months after resection of her right parathyroid glands she was re-hospitalized because of generalized bone pain of 1 month’s duration. A routine urine examination showed: white blood cell 45/hpf (WBC1+), red blood cell 27/hpf (RBC1+), and pH 7. 5. A routine blood test showed: Hb 88 g/L. Her serum potassium was 3.1 mmol/L, calcium 2.83 mmol/L, phosphorus 1.21 mmol/L, CO_2_CP 19.7 mmol/L, creatinine 265 μmol/L, and PTH 1378 pg/ml (Table 1). Her 24-hour urinary calcium was 8.5 mmol/L and 24-hour urinary potassium 29.6 mmol/L. A bone scintigraphy for whole body with ^99m^Tc revealed increased activity in skull, rib, and sternum (Fig. 1c). Parathyroid and thyroid ultrasound showed multiple hypoechoic masses in left lobe, with largest ones being 25 × 23 mm and 22 × 20 mm. She was diagnosed as having MSK and tertiary hyperparathyroidism and underwent left lobe parathyroidectomy on March 27, 2013. A pathological examination of the resected parathyroid tissue showed parathyroid nodular hyperplasia (Fig. 1d). After surgery, her serum calcium was 2.25 mmol/L, phosphorus 1.07 mmol/L, potassium 3.7 mmol/L, CO_2_CP 16.8 mmol/L, PTH 47.81 pg/ml, and creatinine 257 μmol/L (Table 1). Subsequently she was prescribed with oral form of iron and calcitriol at the time of discharge; later, she did not return for the scheduled follow-up visits.

On July 7, 2016, 4 years after parathyroidectomy she was readmitted to our hospital with the complaint of dizziness. A physical examination showed pale appearance, visible thyroid surgery scar, and mild swelling of both lower extremities. A routine urine analysis showed: white blood cell 41/hpf (WBC +) and pH 7.0. A routine blood test showed: Hb 76 g/L, potassium 4.0 mmol/L, calcium 2.09 mmol/L, phosphorus 2.37 mmol/L, CO_2_CP 15.6 mmol/L, serum creatinine 766 μmol/L, and PTH 766.4 pg/ml (Table 1). Renal ultrasound showed MSK, multiple stones in both kidneys, and polycystic kidney disease. She was diagnosed as having MSK and tertiary hyperparathyroidism and underwent renal replacement therapy (hemodialysis). She was started on replacement therapy. At 1-year follow-up, she was undergoing hemodialysis twice a week. A routine blood test showed: Hb 80 g/L. A routine urine analysis showed: PH 7.5 and protein positive (Pro2+). Her serum potassium level was 4.2 mmol/L, calcium 2.22 mmol/L, phosphorus 1.53 mmol/L, CO_2_CP 26.1 mmol/L, creatinine 619 μmol/L, and PTH 546 pg/ml.

**Fig. 2:**
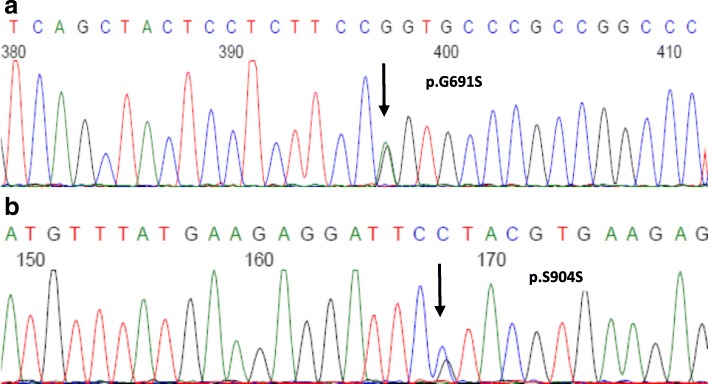
The *RET* gene polymorphism found in the patient with medullary sponge kidney (indicated by an *arrow*). **a** c.2071G>A(G691S) **b** c.2712C>G. (p.S904S)

Given the key role of the GDNF–RET interaction in kidney and urinary tract development, anomalies in these molecules are reasonable candidates for explaining a disorder such as MSK. However, mutations in *RET* proto-oncogene are also present in 97% of individuals with multiple endocrine neoplasia (MEN) 2A (MEN2A). In order to explore the possible genetic mechanisms, we analyzed the *GDNF* and *RET* genes in this patient. After obtaining informed consent from our patient, genomic DNA was extracted from peripheral blood leukocytes by standard phenol–chloroform procedures. All 20 exons and flanking splice sites of the *GDNF* and *RET* genes were amplified by polymerase chain reaction (PCR) with the primers listed in Table 2. Mutations were identified by direct sequencing of PCR products on an ABI 3730xl automated sequencer (Applied Biosystems, USA). Two *RET* polymorphisms were found in this patient, one was nonsynonymous c.2071G>A (G691S; rs1799939) located in exon 11 (Fig. 2a); the other was synonymous c.2712C>G (p.S904S; rs1800863) located in exon 15 (Fig. 2b). No mutation was found in the *GDNF* gene. Both parents of our patient are deceased. Further genetic testing of her husband, daughter, and sister were all negative for this polymorphism.

**Table 2:** Polymerase chain reaction and sequencing primers of *RET* gene

Exon	Sense primer	PCR product (base pairs)	T (°C)
RET-1	5′- GCACCCGCCATCCAGACC -3′	567	62
RET-1	5′- TCTCCTGCCGAAACAGAACTC -3′		
RET-2	5′- CGGCTTGGATGATTGAGAATAGG-3′	583	58
RET-2	5′- GTGATAAGGGCGGCTTGAGG-3′		
RET-3	5’-TCCTCCCCATTCCCGACTG − 3′	573	56
RET-3	5′- AGGCAGGCAAGATTCCAACC-3’		
RET-4	5’-CAACCAGCACGAGTGAGGAC -3’	640	60
RET-4	5’-ACGGAGGCGAAGGACAAATG −3’		
RET-5	5’-AACACACATCTGGTCCACCTATG − 3’	375	56
RET-5	5′- AAGAGCGAGCACCTCATTTCC-3’		
RET-6	5’-GTGTGAAAGTGCGTGTTTGC -3’	428	60
RET-6	5’-CAGTCTACTCTGTGCTGGTTG −3’		
RET-7	5′- GGACTTAGGCTGTGTGGGAATC-3’	516	58
RET-7	5′- -CTGGAAGGAGCAACCATTTACTG-3’		
RET-8	5’-GCTGGTGCTGTTCCCTGTC -3’	375	62
RET-8	5’-CACTGCTGCCCGCTATGC -3’		
RET-9	5’-CCTCCAGTTGCTCCTCCCTAG −3’	470	58
RET-9	5′- CTGCTTCTGAAATCTGTGTGTGC-3’		
RET-10	5’-GGCAGAGTCCTTTGTTCAGATG −3’	452	58
RET-10	5′- CAGCAATTTCCTCCCTTGTTGG-3’		
RET-11	5’-GAGCCTCTGTCTCCATCTGTAAG −3’	606	56
RET-11	5’-CTCGTCTGCCCAGCGTTG −3		
RET-12	5’ GCAGAGACAGGCAGCGTTG--3’	499	58
RET-12	5′- CCTTCCAGGGAGAGCAAAGTC-3’		
RET-13	5′- AGAAGCCTCAAGCAGCATCG-3’	432	58
RET-13	5’-TCAGCCCGTGGACTCAGC-3’		
RET-14	5′- GGCAGAGAGCAAGTGGTTCAAG-3’	618	60
RET-14	5′- GGGCGTGGTGGAGTCAGG-3’		
RET-15	5′- CCACACACCACCCCTCTG-3’	401	58
RET-15	5’-GGCTGAGCGGAGTTCTAATTG −3’		
RET-16	5’-CACTCCTCTGGTTACTGAAAGC -3’	370	56
RET-16	5′- CATTTGCCTCACGAACACATC-3’		
RET-17	5′- CCAGACCCAGGCTGACATC-3’	449	60
RET-17	5′- ATCTACTGCCACACCCAAGG-3’		
RET-18	5′- AGGGTGCGATGGCTGTGG-3’	352	58
RET-18	5′- AGGGTTCAATCTGCTGTCTGC-3’		
RET-19	5’-CTTGGAGAGGTCAGGAGATTGG −3’	453	58
RET-19	5’-AATGCCTAAATGTAAACTGGATGC -3’		
RET-20	5′- TTGGAAACCTGGAACACAAAACC-3’	430	62
RET-20	5′- GCCACCTGGGAACTGAACAC-3’		

*PCR* polymerase chain reaction, *RET* rearranged during transfection, *T* annealing temperature for the PCR reactions

## Discussion

In our study, we present a case of 52-year-old woman with recurrent renal stones, who was subsequently diagnosed as having MSK and tertiary hyperparathyroidism. Although hyperparathyroidism is reported in a few patients with MSK, the exact association is still controversial. Our study indicates that MSK might be the primary factor, and parathyroid adenoma might occur secondary to long-term negative calcium balance caused by MSK, which highlights the importance of early detection, treatment, and further prevention of complications of MSK. Given the key role of the GDNF and RET interaction in kidney and urinary tract development, we also analyzed and found *RET* G691S/S904S polymorphism in this patient, but additional studies are required to explore the exact mechanism of the *RET* gene in MSK with hyperparathyroidism.

MSK is a kidney malformation and is often combined with impaired renal tubular functions, such as concentration defects, and partial or complete distal renal tubular acidosis (dRTA). Fabris *et al.* [4] showed that over 80% of patients with MSK had complete or incomplete dRTA. Chemical analyses of the stone in patients with MSK showed that 67% of them were mainly calcium phosphate and 33% were calcium oxalate which further confirmed the prevalence of distal tubular acidosis in patients with MSK [7]. Some proposed that dRTA played a key role in the pathogenesis of MSK, which could lead to hypercalciuria, hypocitraturia, and stone formation. In our case, our patient had recurrent renal stones, urinary tract infection, hypokalemia, morning urine pH > 5.5, and acidosis, which were consistent with MSK and dRTA. Hyperparathyroidism is also reportedly associated with a few patients with MSK [3, 6], but the exact association between the two diseases is still controversial as both diseases can manifest as kidney stones and excessive urinary calcium excretion. Some scholars believe that hyperparathyroidism is a cause of MSK and triggers nephrolithiasis in these patients [6], while others believe that MSK could be the primary factor, and parathyroid adenoma might occur secondary to long-term negative calcium balance caused by high urinary calcium [4]. In our case, nephrocalcinosis preceded the onset of hyperparathyroidism, while the level of PTH continued to rise after parathyroidectomy. Thus, we hypothesized the tertiary hyperparathyroidism in this patient, as proved by parathyroid pathology, is secondary to MSK, due to long-term distal tubular acidosis and negative calcium balance caused by high urinary calcium.

As MSK is considered a congenital disease, it is speculated that the *GDNF* and *RET* genes, which play a key role in the kidney–urinary tract development and nephrogenesis, might be the reasonable candidates for MSK. Torregrossa *et al.* [8] analyzed *GDNF* and *RET* genes of 55 patients with sporadic MSK and found eight patients with MSK had heterozygous variations of *GDNF* gene c.-45G>C, c.-27 + 18G>A. *RET* encodes a receptor tyrosine kinase, expressed primarily in neural crest and urogenital precursor cells. It is a developmentally important gene, required for kidney morphogenesis, maturation of peripheral nervous system lineages, and for differentiation of spermatogonia. In *RET* knockout mice, the loss of the RET protein results in anomalies of renal embryogenesis, which indicates that the RET protein is required for the development of the urinary excretory system. Primary hyperparathyroidism (PHPT) may occur as part of a complex syndrome or as an isolated disorder. Syndromic PHPT includes MEN types 1 to 4 (MEN1 to MEN4). *RET* gene mutations were also found in 97% of patients with MEN2A, who have an increased risk for parathyroid adenoma or hyperplasia, medullary carcinoma of the thyroid, and pheochromocytoma [9]. Intriguingly, Diouf *et al*. [10] reported the case of a woman with MSK combined with MEN2A, that is, hyperparathyroidism and medullary thyroid carcinoma (MTC), due to *RET* C634Y mutation. In addition, sporadic forms of parathyroid tumors may arise because of somatic gene abnormalities. To date, reports have shown that more than 10% of patients with hyperparathyroidism may have a germline mutation involving the *MEN1*, *RET*, cell division cycle 73 (*CDC73*), calcium-sensing receptor (*CASR*), cyclin-dependent kinase inhibitor (*CDNK1B*), or *PTH* genes. Thakker [11] suggested that the patients with hyperparathyroidism in whom there is a high suspicion of a genetic etiology (for example, young age at onset, multigland disease, parathyroid carcinoma, or atypical parathyroid adenoma) should be offered genetic counseling and germline mutation testing. In our patient, the *RET* gene polymorphisms G691S (exon 11) and S904S (exon 15) were identified, but no *GDNF* variants were found. It was shown that the nonsynonymous *RET* G691S polymorphism is able to increase downstream signaling compared with *RET* wild type [12, 13]. Moreover, some studies have demonstrated that the oncogenic activity of *RET* pathogenic genes in MTC is enhanced by the presence of G691S, suggesting a possible modifier role of this nonsynonymous *RET* polymorphism [14, 15]. Previous studies [16] have shown that the polymorphisms G691S and S904S of *RET* are in linkage disequilibrium with each other. Robledo *et al.* [17] analyzed the polymorphisms G691S and S904S of *RET* in 104 patients with MEN2A, and found the homozygous for these polymorphisms were, on average, 10 years younger at the time of diagnosis compared with heterozygous and wild-type homozygous, indicating that the G691S/S904S variants of *RET* have a modifier effect on the age at onset of MEN2A. However, the exact mechanism of *RET* G691S/S904S polymorphism in the pathogenesis of MSK and hyperparathyroidism still remains to be uncovered.

Considering the pivotal role of incomplete dRTA in MSK, Fabris *et al*. [18] recommended orally administered alkali citrate in patients with MSK with at least one urine abnormality, and found quite positive effects: the stone rate decreased from 0.58 to 0.10 per year, a 50% decrease in their calciuria, and a 75% rise in their citrate levels, with an improvement in their bone mineral density. Other therapeutic measures include drinking plenty of water, reducing dietary sodium and proteins, and increasing vegetable and fruit intake. For patients with very frequently recurring stones, the minimally invasive percutaneous nephrolithotomy can be an option [19]. The progression of MSK is slow and prognosis is usually good. End-stage renal disease in our patient can probably be attributed to persistent kidney damage as a result of long-term uncorrected dRTA, years of undiagnosed hyperparathyroidism, recurrent kidney stones requiring multiple admissions, and repeated episodes of urinary tract infections, all of which complicated the course of this apparently benign disease and resulted in total loss of kidney function. Early detection, treatment, and further prevention of complications will slow the kidney damage and prevent the need of renal replacement therapy in patients with MSK.

## Conclusions

We demonstrated a case of MSK combined with tertiary hyperparathyroidism, which further expands the pathophysiology of this disease. Early recognition of MSK symptoms has important clinical implications for the prevention of further progress of the disease. Besides, we also found *RET* G691S/S904S polymorphism in this patient, but additional studies are required to explore the exact mechanism of the *RET* gene in MSK with hyperparathyroidism.

## Abbreviations

BP: Blood pressure
*CASR*: Calcium-sensing receptor
*CDC73*: Cell division cycle 73
*CDNK1B*: Cyclin-dependent kinase inhibitor
CO_2_CP: Carbon dioxide combining power
CT: Computed tomography
dRTA: Distal renal tubular acidosis
*GDNF*: Glial cell line-derived neurotrophic factor
H: Height
Hb: Hemoglobin
MEN: Multiple endocrine neoplasia
MSK: Medullary sponge kidney
MTC: Medullary thyroid carcinoma
P: Pulse
PCR: Polymerase chain reaction
PHPT: Primary hyperparathyroidism
PTH: Parathyroid hormone
R: Respiratory rate
RET: Rearranged during transfection
RR: Reference range
T: Temperature
W: Weight
